# The effects of a commercial liquid energy supplement on physical performance, reaction time, and mood state in college-aged males and females

**DOI:** 10.1186/1550-2783-10-S1-P5

**Published:** 2013-12-06

**Authors:** J Jay Dawes, Jasmine Richmond, Don Melrose, Liette Ocker, Steve W Edwards, Kelly A Brooks, Dianea Willis

**Affiliations:** 1 Texas A&M University- Corpus Christi, TX, USA; 2 Texas A&M University, TX, USA; 3 Sam Houston State University, TX, USA; 4 Oklahoma State University, OH, USA

## Background

Consumption of caffeine-containing liquid energy supplements has increased dramatically over the past several years. Many of these products are marketed toward individuals seeking to boost energy and arousal levels. Consequently, many active individuals consume energy drinks hoping to improve time to fatigue, increase work capacity and facilitate faster training adaptations.

## Purpose

The purpose of this study was to investigate the effects of a commercial energy supplement on physical performance, reaction time and mood state in college-aged students.

## Methods

Nineteen subjects (n=19; 8 male, 11 female; age 22.42 ± 3.15 years; body mass: 68.95 ± 12.70 kg; BMI: 23.86 ± 2.85; ht: 168.7 cm) volunteered to participate in the study. All test subjects completed a health history and medical questionnaire, as well as an informed consent form, prior to participation. Participants completed pre and post-testing, consisting of the following: Profile of Mood States (POMS) Questionnaire, countermovement vertical jump, YMCA Bench press test, sit-ups to fatigue, Dynavision™ reaction test, and a 20-meter sprint repeat test. The pre and post-test sessions were conducted with a period of 48 hours between. Thirty minutes prior to post-testing, subjects ingested a serving (2oz) of the pre-exercise energy supplement (Redline Powershot by VPX) or a placebo. Administration of the supplement was double blind. Ten (n=10) participants received the supplement, while nine (n=9) participants received the placebo. A paired samples t-test was used to determine between group differences for the selected assessments, at an alpha level of 0.05.

## Results

Data analysis indicated a significant interaction between the treatment effect and the participants sit-up to fatigue scores, *t* (9) = 0.80, *p* ≤ 0.05. Further examination of posttest main effects revealed a significant difference between pre and posttest scores on the Dynavision™ reaction test for both the placebo, t(8) = -3.12, *p* ≤ 0.01, and the treatment *t* (9) = -2.92, *p* ≤ 0.05. This represented a 13.40% increase in the treatment group’s posttest sit-up score, compared to an 11.89% decrease in the placebo group’s score. Additionally, the treatment group improved 3.4% on their Dynavision™ reaction test posttest score, while the placebo group only improved 2.56 %. While POMS data revealed no significant difference, there appears to be a strong positive trend among those who received the treatment when compared to participants receiving the placebo.

## Discussion

A caffeine-containing, liquid energy supplement may improve time to fatigue on endurance assessment for the trunk musculature. While no significance was discovered between the treatment and placebo group for POMs scores, the data suggests a strong positive trend for those that consumed the treatment when compared to the placebo. These findings warrant further investigation.

**Figure 1:**
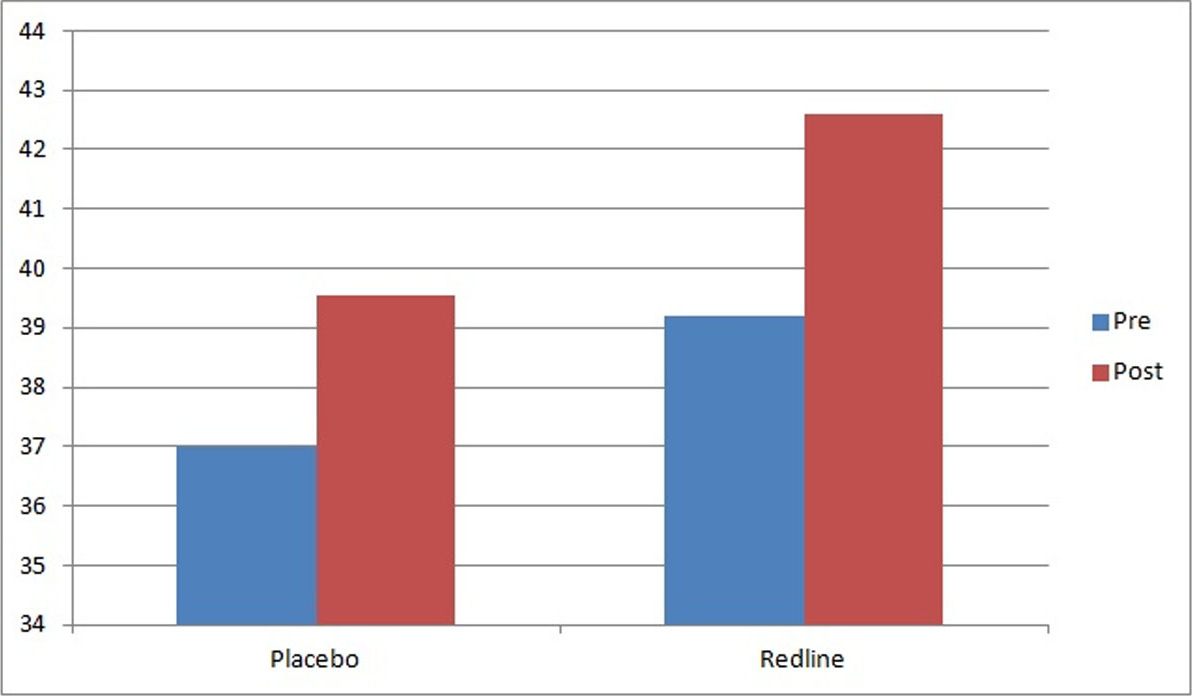
Results for D2 Reaction Test

**Figure 2:**
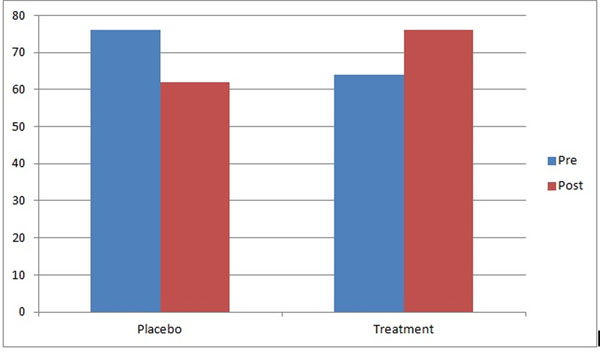
Results for Sit-ups to Fatigue

